# Classification models for clear cell renal carcinoma stage progression, based on tumor RNAseq expression trained supervised machine learning algorithms

**DOI:** 10.1186/1753-6561-8-S6-S2

**Published:** 2014-10-13

**Authors:** Zeenia Jagga, Dinesh Gupta

**Affiliations:** 1 Bioinformatics Laboratory, Structural and Computational Biology Group, International Centre for Genetic Engineering and Biotechnology (ICGEB), Aruna Asaf Ali Marg, New Delhi, India

## Abstract

**Background:**

Clear-cell Renal Cell Carcinoma (ccRCC) is the most- prevalent, chemotherapy resistant and lethal adult kidney cancer. There is a need for novel diagnostic and prognostic biomarkers for ccRCC, due to its heterogeneous molecular profiles and asymptomatic early stage. This study aims to develop classification models to distinguish early stage and late stage of ccRCC based on gene expression profiles. We employed supervised learning algorithms- J48, Random Forest, SMO and Naïve Bayes; with enriched model learning by fast correlation based feature selection to develop classification models trained on sequencing based gene expression data of RNA*seq *experiments, obtained from The Cancer Genome Atlas.

**Results:**

Different models developed in the study were evaluated on the basis of 10 fold cross validations and independent dataset testing. Random Forest based prediction model performed best amongst the models developed in the study, with a sensitivity of 89%, accuracy of 77% and area under Receivers Operating Curve of 0.8.

**Conclusions:**

We anticipate that the prioritized subset of 62 genes and prediction models developed in this study will aid experimental oncologists to expedite understanding of the molecular mechanisms of stage progression and discovery of prognostic factors for ccRCC tumors.

## Background

Renal cell carcinoma is a common adult kidney cancer, accounting for 2-3% of all new cancer cases diagnosed worldwide [1]. Detection of renal cell carcinoma at an early stage is difficult and generally diagnosed incidentally [2]. Most cases can be treated effectively only if detected timely, increasing the survival rates of patients [3]. The clear-cell Renal Cell Carcinoma (ccRCC) is the most common subtype of the renal cell carcinoma, characterized by clear cell morphology of the cytoplasm [4]. So far, early stage diagnosis is difficult due to the molecular complexity and divergent clinical behavior of ccRCC patients [5]. Hence, there is an urgent need to determine candidate biomarkers for diagnosis and/or prognosis for stage specific distinction in ccRCC.

Advancements in high throughput technologies like Next Generation Sequencing have opened novel avenues in cancer research with renewed emphasis on diagnosis, prognosis and therapeutics. The launch of large scale comprehensive multi-node programs like International Cancer Genomic consortium [ICGC] (http://icgc.org/) and The Cancer Genome Atlas [TCGA] (http://cancergenome.nih.gov/) enables systematic studies on genomic, epigenomic and transcriptomic levels for different cancer types that hold clinical and societal importance globally [6]. These projects are making data available to researchers in different levels 1, 2, 3, 4 (i.e. raw, processed, interpreted and summarized respectively) enabling genome informed personalized cancer medicine research [7,8].

Along with advances and affordability of high throughput technologies and data, progress is also being made towards personalized and predictive medicine for clinical management of cancer patients. In order to analyze diverse and multidimensional cancer related data, machine learning techniques are being extensively applied for cancer prognosis as well as diagnosis [9,10].

In ccRCC, clinical tumor staging by TNM staging system is used - as prognostic factor; determines treatment regimen of patient; confirmed to guide the surveillance protocols and to assess risk of metastatic renal cell carcinoma [3,11-13]. Although tumor stage being an effective prognostic factor, to our knowledge there has been no systematic studies characterizing gene expression data for tumor stage progression. Thereby, we hypothesized that identifying gene expression signature that correlates with clinical tumor stage progression might lead to discovery of a panel of prognostic molecular signatures for ccRCC tumors.

In this study, we have developed prediction models to discriminate clinical tumor stages- early stage (I, II) and late stage (III, IV) of ccRCC. The prediction models are trained on gene expression data of RNA*seq *experiments from TCGA by implementing state-of-art supervised machine learning algorithms. The gene expression signatures identified by feature selection approach, which enriched classifier training, helped us to efficiently classify the tumors based on their clinical tumor stage. Further, we found that amongst the implemented machine learning techniques, predictive models based on Random Forest algorithm performed the best with accuracy of 76.84% on independent data test, area under Receiver Operating Characteristic Curve (auROC) of 0.778.

## Methods

**Figure 1:**
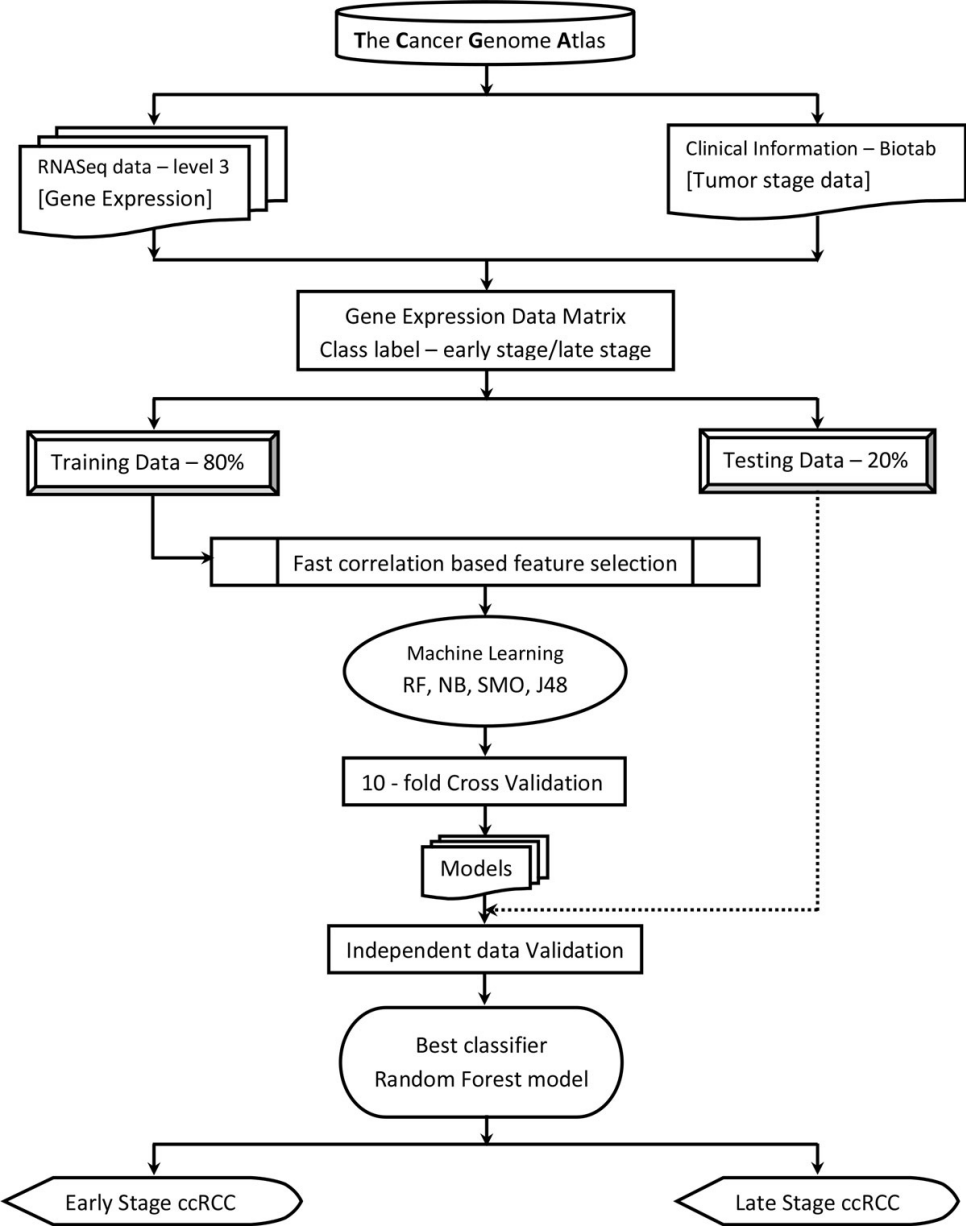
**Schematic representation of the overall strategy used in the study**. Data was downloaded from TCGA data portal. Gene expression values of the tumors constituted the descriptor values. Class labels were derived from the clinical information given in the Biotab of the TCGA data portal. Resulting data was processed to generate training/testing data files. Feature selection and generation of classification models was performed on WEKA Workbench.

The overall methodology followed in the article is summarized in Figure 1.

### Data mining

The dataset for this study was downloaded from TCGA data portal (https://tcga-data.nci.nih.gov/tcga/tcgaHome2.jsp). The Level 3 RNAseq expression dataset from cancer type KIRC i.e. Kidney Renal clear cell carcinoma on Illumina HiSeq 2000 RNA sequencing platform was obtained from TCGA data portal [14]. The RNAseq expression data was available for 475 tumor samples. Each patient gene expression profile consists of gene expression data for 20,534 genes. Only tumor samples were taken into account in the study. The clinical information for selected subjects was retrieved from the "clinical Biotab" section of the data matrix based on the BCR (Biospecimen Core Resource) IDs of the patients.

We generated a gene expression data matrix in Comma Separated Value (CSV) file format from the data retrieved from the TCGA data portal, with 20,534 genes as column labels and 475 patients BCR ids as row labels. This was achieved using in-house shell scripts. The 'Reads Per Kilobase per Million' i.e. RPKM values of the mapped reads, retrieved as an estimate for gene expression, were used as feature vectors for classifiers trainings. In order to generate a study dataset, we marked class label of "Early Stage" for patients with clinical tumor stage I & II, and class labels of "Late Stage" for tumor stage III & IV.

Testing and Training dataset gene expression matrix with 9756650 (20,534 × 475) data points was randomly stratified and split into 80% training-cum-validation dataset (Additional File 2) and 20% independent testing dataset (Additional File 3) using a PERL script, developed in house.

### Machine learning

All the Machine learning steps including data pre-processing, feature selection, generating classification models and independent testing was performed on Waikato Environment for Knowledge Analysis (WEKA) version 3.7.9 [15].

*Feature selection: *For reducing the dimensionality in feature space and improving instance to feature ratio for better machine learning, we combined WEKA attribute evaluator 'SymmetricalUncertAttributeSetEval' with search method of 'FCBFSearch'. The algorithm Fast Correlation Based Feature (FCBF) selection utilizes predominant correlation to identify relevant features in high dimensional datasets [16]. This feature selection method is used in conjunction with evaluator 'SymmetricalUncertAttributeEval', which selects subset of features based on symmetrical uncertainty with respect to another features [16,17].

We then analyzed this subset gene list for Gene Ontology (GO)- biological process annotations with WebGestalt (http://bioinfo.vanderbilt.edu/webgestalt/), a Web-based Gene Set Analysis Toolkit [18]. We visualized the impact of our candidate genes at genomic (mutation, copy number alterations), transcriptomic (expression) and protein levels (expression) in ccRCC TCGA dataset through cBio Cancer Genomics Portal [19,20]. We also estimated overall Kaplan-Meier Survival curve based on cases with and without alteration in these genes using cBio Cancer Genomics Portal.

### Classification algorithms

We employed four different state-of-the-art supervised machine learning algorithms (J48, Naïve Bayes, Sequential minimal optimization and Random Forest) for generating the classification models. J48 is the java based implementation decision tree learning algorithm C4.5 [21]. Naïve Bayes algorithm works on the assumption that all the features are statistically independent and is based on Bayes theorem [22]. Sequential Minimal Optimization (SMO) algorithm is an implementation of Support Vector Classifier [23,24]. Random Forest is randomly constructed ensemble of independent decision trees [25].

*Training-cum-validation: *The four supervised machine learning algorithms (J48, LibSVM, Naïve Bayes, and Random Forest) were trained on the subset features from feature selection and further validated by 10 fold cross-validation. The training models thus generated were compared based on the accuracy and auROC. Random Forest classifier was optimized for number of trees and the models were generated using number of trees as 1000.

*Independent dataset test*: Independent data testing is recommended to exclude the "memory" effect or bias for trained classification models [26]. So, we re-evaluated the performance of best-trained and cross-validated model on independent dataset.

### Performance evaluation

In order to evaluate the generated prediction models we employed various evaluation metrics recommended for evaluating the classifier's performances i.e. accuracy, sensitivity or recall, specificity, Matthews Correlation Coefficient (MCC), F-value and auROC [27,28]. The auROC is area under curve and is evaluated for comparing performance of several classifiers.

## Results

### Gene expression data matrix

The RNAseq dataset for 475 clinically diagnosed ccRCC patients were retrieved from TCGA data portal. The dataset includes gene expression levels for 20,534 genes in tumor tissue samples of the patients. Thus, we generated a study dataset of gene expression data matrix of 20,534 × 475, columns and rows respectively. The data characteristics of patients whose gene expression values have been used in this study are given as Additional File 1. Further, the study dataset was divided into 80% training-cum-validation and 20% independent testing dataset. The distribution of patients across training and testing dataset by clinical tumor stage is given in Table 1.

**Table 1:** Summary of Training and Testing dataset statistics.

Class label	Clinical status	Training	Testing
**Early Stage**	Stage I	180	45
**Early Stage**	Stage II	39	10
**Late Stage**	Stage III	97	24
**Late Stage**	Stage IV	64	16
		**380**	**95**

### Feature selection

Feature selection performed on training-cum-validation dataset of 219 early stage and 161 late stage instances by fast correlation based feature selection largely reduced the feature vectors space from 20,534 genes to 62 genes (see additional files: 4 for list of genes, 5 for distribution plot of the expression values for all the selected 62 genes). From the GO Slim Annotation of subset genes, we identified "multicellular organismal process" (29 genes), "response to stimulus" (28 genes), "metabolic process" (26 genes) and "biological regulation" (26 genes) as most frequent GO annotations for Biological Processes (Additional file 6). Oncoprint of the 62 identified candidate gene list by feature selection determined the alterations of genomic profiles in 83.6% of ccRCC cases (Additional file 6). Further validation of the relevance of selected gene features was achieved by performing survival Kaplan-Meir estimate. The survival estimate revealed that the median months survival in cases with alterations is 73.17 months and cases without alterations is 90.38 months (Additional File 6).

### Training-cum-validation

We evaluated training models by 10 fold cross-validation for the classifiers trained on the four supervised machine learning algorithms- SMO, Random Forest, J48 and Naïve Bayes. All the models were based on standard error base classifiers. The performances of the generated prediction models were compared on the basis of accuracy and auROC values (Table 2). The classification accuracy of the generated prediction models ranges from 67.6% for J48, to 79.7% for Random Forest; and auROC value ranges from 0.7 for J48 to 0.876 for Random Forest. Based on accuracy and auROC, we inferred that Random Forest based prediction model has outperformed other three machine learning algorithms implemented in the study.

**Table 2:** Performance of prediction models generated by 10-fold cross validation from training-cum validation dataset.

Classifier	Accuracy	auROC^#^
**J48**	67.6316	0.700
**Naïve Bayes**	77.8947	0.843
**Random Forest**	**79.7368**	**0.876**
**SMO**	76.0526	0.745

# auROC denotes area under Receivers Operating Characteristic curve. Highest numerical value in each column is highlighted as bold letters.

### Independent data testing

Furthermore, we evaluated our prediction models on an independent dataset with 55 early stage patients and 40 late stage patients. The performances of the prediction models were compared on the basis of standard statistical measures- accuracy, sensitivity, specificity, F-measure, and auROC (Table 3). We observed coherence in the performance of the models between independent data testing and 10 fold cross validation based on auROC values.

**Table 3:** Performance of prediction models by standard statistical evaluation parameters for independent testing dataset.

Classifier	Accuracy	Sensitivity	Specificity	F-value	auROC^#^
**J48**	62.11	79.63%	39.02%	0.704918	0.563
**Naïve Bayes**	72.63	**93.56%**	48.78%	0.790323	0.749
**Random Forest**	**76.84**	88.89%	**60.98%**	**0.813559**	**0.778**
**SMO**	73.68	87.04%	56.10%	0.789916	0.716

# auROC denotes area under Receivers Operating Characteristic curve. Highest numerical value in each column is highlighted as bold letters.

**Table 4:** Literature validation of the genes selected by feature selection

Category	Genes
**Renal Cancer**	MAPK7, FGFR3, OASL, GUCY2D, GHRH
**Other Renal Disorder**	NOZ2, APOL1
**Cancer Progression in other cancers**	IRF7, FOXA1, GREB1L, TOB1, RTP3, IER2, RORL
**Biomarker in other cancers**	SHOX, HDGFL1, HUS1B, GNG7, AP1M1

**# **Detailed information about the genes selected by feature selection can be found in Additional File 4

**Figure 2:**
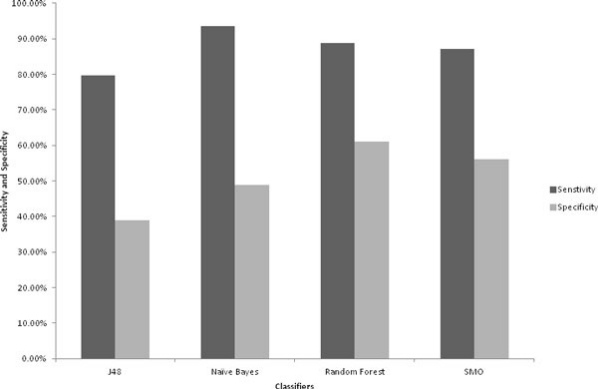
**Sensitivity - Specificity plots**. The sensitivity - specificity plot for the classifiers was analyzed to reveal optimal prediction of the models. All the generated prediction models had higher sensitivity than specificity. Random Forest based classifier was ranked highest with optimal performance in sensitivity and specificity.

We evaluated sensitivity and specificity plot to determine prediction model with low error rates i.e. high sensitivity and specificity. Sensitivity of all the models was in the range of 79-94%, with highest sensitivity of 93.56% for Naïve Bayes. Specificity of the models varied in a wide range with lowest of 39.0% for J48 and the highest of 60.98% for Random Forest (Figure 2). Although, Naïve Bayes based model shows the highest sensitivity, the best sensitivity-specificity trade-off was observed for Random Forest Classifier with 88.89% sensitivity and 60.98% specificity.

The prediction accuracies of all the four predictive models on independent dataset were- 62.11, 72.63, 76.84, 73.68 for J48, Naive Bayes, Random Forest and SMO respectively (Table 3). F-measures of the models developed in the study is between 0.7 to 0.82. It is notable that amongst the four evaluated prediction models, the model based on Random Forest displays highest accuracy and F-measure.

**Figure 3:**
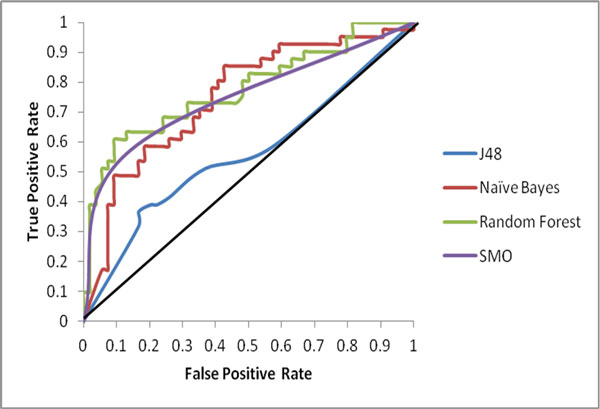
**ROC plot**. Receivers Operating Characteristic curve (ROC) plot all the four classifiers. Amongst all the prediction models, Random Forest achieved the maximum value for area under Receivers Operating Characteristic curve (auROC) closely followed by Naïve Bayes and SMO. J48 had the least auROC. Scalar values of auROC are given in Table 3.

ROC plot is evaluated to characterize threshold independent performance of the prediction models. The ROC plot of the classifiers showing tradeoff between true positive rate and false positive rate in Figure 3 and auROC values in Table 3 suggests that prediction models based on Random Forest algorithm performed better than Naïve Bayes, SMO and J48. The prediction models had an auROC value of more than 0.5 i.e. better than random predictions, assuring their robust performance.

Thus, on the basis of statistical evaluators used for characterizing the performance of the prediction models, classifiers were ranked in following order- Random Forest, Naïve Bayes, SMO and J48.

Hence, Random Forest based prediction model is an efficient classifer with 88.89% sensitivity, 76.84% accuracy and auROC of 0.778 for classifying early stage and late stage of the ccRCC tumors, using gene expression profiles.

## Discussion

Predictive classification models in cancer biology are employed with either of the 3 foci- predicting cancer susceptibility, recurrence or survivability [9]. Previous studies in ccRCC employs gene expression profiles to predict survival [29,30] and determine newer tumor subtypes [31,32]. We have used gene expression profiles of ccRCC stage specific tumor tissues to train supervised machine learning algorithms, towards our goal to develop classification models for early stage to late stage tumor progression of ccRCC.

The TCGA gene expression data for ccRCC patients has diverse representation of age, gender and tumor stage (Additional File 1). The patients with localized tumors (stage I and II) were class labeled as early stage whereas patients with locally advanced disease (stage III) and metastatic disease (stage IV) as late stage. The TCGA gene expression profile data available for 475 tumor samples and 20,534 genes had no significant batch effects [14] and came with the "curse of dimensionality". An efficient fast filter feature selection method based on predominant correlation (fast correlation based feature selection) largely reduced the feature space from 20,534 genes to 62 gene expression profiles.

To analyze the details of selected training set genes used in development of prediction models for ccRCC, we first performed GO annotations in which the corresponding biological processes displayed very broad but consistency with cancer hallmarks [33]. Secondly, the oncoprint visualization of the selected subset genes displayed alterations in all the genomic profiles in 83.6% of the TCGA ccRCC cases. Thirdly, ccRCC cases with alterations in the selected genes had better overall survival than cases without alteration in these genes. Lastly, the literature mining of the selected genes revealed that 42 out of 62 genes are already reported in literature for its involvement in cancer progression or detection, either ccRCC or any other type of cancer. This indeed increased our confidence on the applicability of 62 selected genes for development of prediction model as well as the prioritized list to be analyzed for therapeutic and prognostic potential in case of ccRCC.

We found that the prediction model based on Random Forest algorithm is the best classifier followed by Naïve Bayes and SMO, which performed reasonably better than J48 based prediction model. The results demonstrate that early stage and late stage ccRCC tumors can be classified with high sensitivity using gene expression profiles. Our observations re-emphasizes the fact that machine learning based models will play important role in the developing field of predictive and personalized medicine.

Our study also illustrates a new method for classification in ccRCC, wherein tumor stage information can be derived from the molecular features, instead of tumor size. In future, availability of additional ccRCC tumor patients and inclusion of more feature vectors like miRNA expressions, protein expression and SNP profiling data could further enhance the accuracy of the prediction models given the fact that ccRCC tumors typically have heterogeneous molecular profiles. Though we tested the prediction models on independent testing datasets of 95 patients, the outcome of such models needs to be interpreted judiciously before incorporation into a clinical set up. We anticipate that the selected gene expression features and the prediction models developed in this study would expedite the challenge for discovering molecular prognosis factors and stage progression molecular signatures in ccRCC tumors.

## Conclusion

An empirical approach has been employed in this study to develop classification model for tumor stage progression in ccRCC based on gene expression profiles. We have identified a subset of 62 genes by feature selection, the expression profiles of which predominantly correlates with tumor-stage of the patient given the heterogeneous data in terms of tumor stage, age group and gender of the patient. We report that Random Forest based prediction model accurately and reliably classifies patient tumor stages. We anticipate that such prediction models based on the molecular correlates could contribute to the optimal management of patients in ccRCC. Currently, the prediction models generated are available upon request to the authors. To the best of our knowledge, we are reporting the first prediction model to classify ccRCC tumor stage based on gene expression profiles.

## Competing interests

The authors declare that they have no competing interests.

## Declarations

Publication of this work was funded by International Centre for Genetic Engineering and Biotechnology, New Delhi, India.

## Authors' contributions

ZJ and DG designed the study. ZJ implemented the study. ZJ and DG have prepared the manuscript and approve the manuscript.

## Supplementary Material

Additional file 1 **Data characteristics of ccRCC level 3 information for TCGA patients for KIRC. This file consists of 2 figures - Gender wise stage distribution of the TCGA patients; and Age distribution of the patients**. The figures are in a Portable Document Format (PDF) and can be viewed with any standard PDF viewer. Click here for file

Additional file 2 **Training dataset. This file consists of Training dataset used in this study**. The file is in a Comma Separated Value (CSV) format with 20,534 gene's expression in RPKM in column and 380 patients as rows. The file is in CSV format. Click here for file

Additional file 3 **Testing dataset. This file consists of Testing dataset used in this study**. The file is in a Comma Separated Value (CSV) format with 20,534 gene's expression in RPKM in column and 95 patients as rows. The file is in CSV format. Click here for file

Additional file 4 **Gene symbols and gene names and literature validation of the selected genes after feature selection.** This file consists of 62 genes selected by feature selection in the study and gene names annotated by DAVID-gene name batch viewer (http://david.abcc.ncifcrf.gov/). Literature validation of 42 out of 62 genes for involvement in renal cancer, renal disease, including disease association for cancer progression, & biomarkers in other cancers is given from literature and Gene Cards (http://www.genecards.org/). The file is in .docx format and can be viewed using any document viewer like Microsoft Word. Click here for file

Additional file 5 **Distribution plot of the expression values selected 62 genes.** This file consists of expression value distribution plots generated by Weka Explorer of final 62 genes for the class label "Early Stage" and "Late Stage". The file is in .docx format and can be viewed using any document viewer like Microsoft Word. Click here for file

Additional file 6 **Analysis of the 62 selected genes for GO annotations - Biological Process; Oncoprint and Overall Survival Kaplan-Meier Estimate from cBioportal for Cancer Genomics.** This file consists of 3 figures - Bar chart of GO biological process categories of the selected 62 genes; Screen Shot of Oncoprint for genomic alterations in selected 62 genes in ccRCC cases in cBioPortal for Cancer Genomics age distribution of the patients; and Survival Kaplan-Meier Estimate for selected 62 genes in ccRCC cases in cBioPortal for Cancer Genomics. The figures are in a Portable Document Format (PDF) and can be viewed with any standard PDF viewer. Click here for file
