# Supplementary material for: Classification models for clear cell renal carcinoma stage progression, based on tumor RNAseq expression trained supervised machine learning algorithms
Source: BMC Proc. 2014 Oct 13;8(Suppl 6):S2. doi: 10.1186/1753-6561-8-S6-S2 (PMC4202178; doi:10.1186/1753-6561-8-S6-S2)

Figure S1A. Gender wise stage distribution of the TCGA ccRCC patients

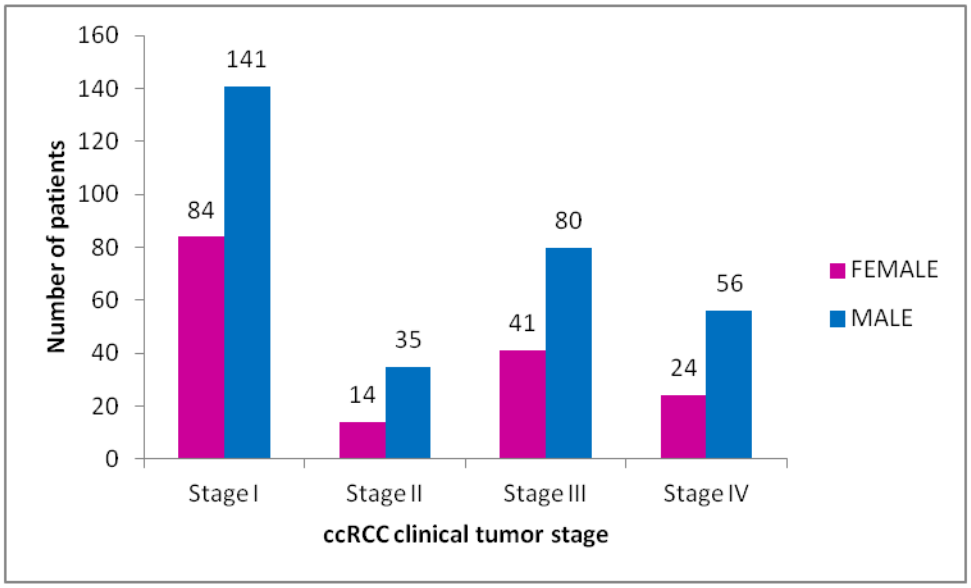

Figure S1B. Age distribution of the TCGA ccRCC patients

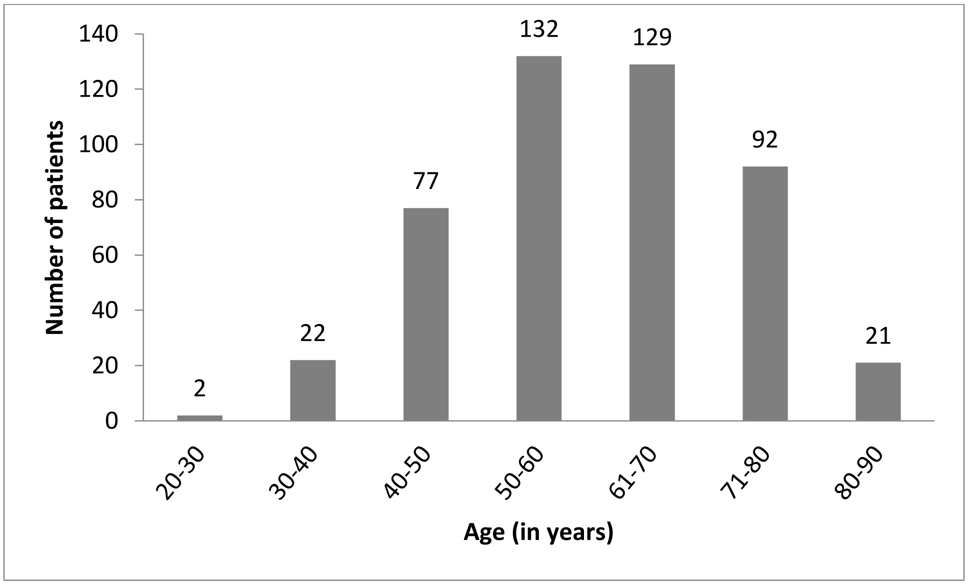

Supplement: Additional file 1 — Data characteristics of ccRCC level 3 information for TCGA patients for KIRC. This file consists of 2 figures - Gender wise stage distribution of the TCGA patients; and Age distribution of the patients. The figures are in a Portable Document Format (PDF) and can be viewed with any standard PDF viewer. [file 1753-6561-8-S6-S2-S1.pdf]
