# Supplementary material for: Classification models for clear cell renal carcinoma stage progression, based on tumor RNAseq expression trained supervised machine learning algorithms
Source: BMC Proc. 2014 Oct 13;8(Suppl 6):S2. doi: 10.1186/1753-6561-8-S6-S2 (PMC4202178; doi:10.1186/1753-6561-8-S6-S2)
Supplement: Additional file 4 — Gene symbols and gene names and literature validation of the selected genes after feature selection. This file consists of 62 genes selected by feature selection in the study and gene names annotated by DAVID-gene name batch viewer (http://david.abcc.ncifcrf.gov/). Literature validation of 42 out of 62 genes for involvement in renal cancer, renal disease, including disease association for cancer progression, & biomarkers in other cancers is given from literature and Gene Cards (http://www.genecards.org/). The file is in .docx format and can be viewed using any document viewer like Microsoft Word. [file 1753-6561-8-S6-S2-S4.docx]

| S.No. | Gene Symbol | Gene Name | Associated Cancer Type | Remarks and References |
| --- | --- | --- | --- | --- |
|  | MAPK7 | Mitogen Activated Protein Kinase 7 | Renal clear cell carcinoma  Pancreatic cancer | -promotes cell growth and proliferation in response to tyrosine kinase signaling [[1](#_ENREF_1)]  -target gene for miR-143 which is upregulated in pancreatic cancer and ccRCC [[2](#_ENREF_2)] |
|  | FGFR3 | fibroblast growth factor receptor 3 | Renal clear cell carcinoma | Reported to be differentially up-regulated in ccRCC patient [[3](#_ENREF_3), [4](#_ENREF_4)] |
|  | OASL | 2'-5'-oligoadenylate synthetase-like | Renal cell carcinoma | Transcript level upregulated by an average of 50% or more n high CES-D patients. [[5](#_ENREF_5)] |
|  | GUCY2D | guanylate cyclase 2D, membrane (retina-specific) | Renal cell carcinoma | Involved in Somatostatin anti-apoptosis pathway , reported to be enriched in genome wide CpG island methylation analysis in pathogenesis of renal cell carcinoma [[6](#_ENREF_6)] |
|  | GHRH | growth hormone-releasing hormone. | Renal cell carcinoma | -reported to be expressed in various tumors [[7](#_ENREF_7)]  -Renal cell carcinoma reported to express distinct binding site for growth hormone-releasing hormone [[8](#_ENREF_8)] |
|  | EPB49 | erythrocyte membrane protein band 4.9 | Prostate Carcinoma | Reported in top frequently selected gene in leave-one-out cross validation using gene expression in prostate carcinomas during modeling [[9](#_ENREF_9)] |
|  | GREB1L | Growth regulation by estrogen in breast cancer-like | Prostate cancer, Breast cancer | -reported as novel androgen-regulated gene required for prostate cancer growth [[10](#_ENREF_10)]  -reported as estrogen-regulated genes expressed in hormone-responsive breast cancer [[11](#_ENREF_11)] |
|  | EIF4EBP1 | EIF4E Binding Protein 1 | Prostate cancer | Hypothesized putative target of miRNA-175B; Increased expression in prostate cancer tissues [[12](#_ENREF_12)] |
|  | P704P | prostate-specific P704P | Prostate Cancer | Reported to part of genomic cluster of prostate-specific genes [[13](#_ENREF_13)] |
|  | PAR4 | Protease-activated receptor 4 | Prostate Cancer | -reported to be over-expressed in prostate cancer [[14](#_ENREF_14)] |
|  | GPX3 | Glutathione Peroxidase 3 | Ovary clear cell carcinoma,  Prostate cancer | -highly expressed in clear cell carcinoma of ovary and involved in detoxification[[15](#_ENREF_15)]  -Novel tumor suppressor gene reported in prostate cancer [[16](#_ENREF_16)] |
|  | TOB1 | transducer of ERBB2, 1 | Ovarian cancer, Breast cancer | -Progression free survival indicated by long survival associated with hypomethylation at TOB1 CpG sites in Ovarian cancer [[17](#_ENREF_17)]  - high TOB1 expression in a cohort of node-negative patients demonstrated significantly shortened distant metastasis-free survival for patients with in Breast cancer. [[18](#_ENREF_18)] |
|  | DNASE1L3 | deoxyribonuclease I-like 3 | Ovarian cancer | For targeted therapy in ovarian cancer [[19](#_ENREF_19)] |
|  | MPZL2 | myelin protein zero-like 2 | Ovarian cancer | Reported to be upregulated in ovarian cancer [[20](#_ENREF_20)] |
|  | NCRNA00051 | non-protein coding RNA 51 | Ovarian cancer | Reported to be showing copy number variation - amplification in BRCA1 ovarian cancer patients [[21](#_ENREF_21)] |
|  | RPS11 | Ribosomal Protein S11 | OsteoSarcoma | (Gene Cards) |
|  | ALX1 | ALX homeobox 1 | Non small cell lung cancer | Reported as part of the DNA methylation signature for Stage I Non small cell lung cancer [[22](#_ENREF_22)] |
|  | COL7A1 | collagen, type VII, alpha 1 | Meningiomas | -Cell adhesion  -Upregulated with a fold change of >=2 in grade 2 and 3 meningomas as compared to grade 1 [[23](#_ENREF_23)] |
|  | SHOX2 | short stature homeobox gene | Lung cancer,  Breast cancer | -Dna methylation of SHOX2 is identified as a diagnostic biomarker for lung cancer[[24](#_ENREF_24)]  -reported to overrepresented in breast cancer [[25](#_ENREF_25)] |
|  | NOD2 | Nucleotide-binding oligomerization domain-containing protein 2 | Hyperhomocysteinemia | Reported to ameliorate renal injury in mice with Hyperhomocysteinemia (independent risk factor in the progression of end-stage renal disease)[[26](#_ENREF_26)] |
|  | CACNG6 | calcium channel, voltage-dependent, gamma subunit 6 | Hepatocellular Carcinoma | reported to be differentially expressed common gene in primary cultured invasive phenotype hepatocellular carcinoma cells [[27](#_ENREF_27)] |
|  | RTP3 | receptor (chemosensory) transporter protein 3 | Hepatocellular carcinoma | Also referred as TMEM7 ; reported to suppress cell proliferation and is down- regulated in hepatocellular carcinoma. [[28](#_ENREF_28)] |
|  | SLC35E3 | Solute carrier family 35,member E3 | Glioblastoma multiforme tumors | -reported as a novel gene, over expressing in glioblastoma multiforme tumors [[29](#_ENREF_29)] |
|  | NKX2-2 | NK2 homeobox 2 | Ewing’s sarcoma,  [Gastrointestinal neuroendocrine tumor](http://www.malacards.org/card/gastrointestinal_neuroendocrine_tumor) | -Reported as critical target gene in Ewing’s sarcoma [[30](#_ENREF_30)]  - Acts as a transcriptional activator, associated disease as [Gastrointestinal neuroendocrine tumor](http://www.malacards.org/card/gastrointestinal_neuroendocrine_tumor) (Gene Cards) |
|  | RSPO2 | R-spondin 2 | Colorectal cancer | -reported to have positive role for tumor growth based on FOXQ1 overexpression in colorectal cancer [[31](#_ENREF_31)] |
|  | HUS1B | \| HUS1 (S. Pombe) Checkpoint Homolog Checkpoint Homolog B \| \| --- \| \|  \| | Colorectal Cancer | in the panel of genetic colorectal cancer markers [[32](#_ENREF_32)] |
|  | IER2 | Immediate early response-2 | Colorectal cancer | Reported to promote tumor cell motility and metastasis and predict poor survival of colorectal cancer patients [[33](#_ENREF_33)] |
|  | TBX18 | T-box transcription factor | Colon Cancer | -Transcription factor  -reported for tumor-specific methylation in primary colon cancer [[34](#_ENREF_34)] |
|  | HDGFL1 | Hepatoma derived growth factor-like 1 | Colon cancer | -in the panel of biomarkers for early detection and prognosis of colon cancer [[35](#_ENREF_35)] |
|  | SLC22A16 | solute carrier family 22 (organic cation/carnitine transporter), member 16 | [clear cell adenocarcinoma](http://www.malacards.org/card/clear_cell_adenocarcinoma) | -High affinity carnitine transporter (Gene Cards) |
|  | APOL1 | Aplolipoprotein L1 | Chronic Renal failure | reported to be involved in chronic progressive renal failure [[36](#_ENREF_36)] |
|  | SUMO4 | Small Ubiquitin-Like Modifier 4 | Carcinogenesis | One of 4 isoforms of Small Ubiquitin-like modifier present in human; important cell cycle protein, implicated in cancer development [[37](#_ENREF_37)] |
|  | EIF5B | Eukaryotic translation initiation factor 5B | Cancer | - reported that eIFs can act as oncogenes or tumor suppressors [[38](#_ENREF_38)] |
|  | KCNAB1 | potassium voltage-gated channel, shaker-related subfamily, beta member 1 | Cancer | Encodes accessory beta-subunit of the core potassium ion channel which is implicated as potassium ion channel in cancer [[39](#_ENREF_39)] |
|  | FOXA1 | Forkhead-box A1 | Breast cancer, Prostate cancer,  Bladder cancer | -reported to promote tumor progression in prostate cancer [[40](#_ENREF_40)]  -associated with high grade, late stage bladder and increased tumor proliferation [[41](#_ENREF_41)]  -associated with prognostic significance in breast cancer [[42](#_ENREF_42)] |
|  | IRF7 | interferon regulatory factor 7 | Breast cancer | Involved in breast cancer progression [[43](#_ENREF_43), [44](#_ENREF_44)] |
|  | GNG7 | guanine nucleotide binding protein (G protein), gamma 7 | Breast Cancer | One of the top gene marker for discriminating low and high grade tumors  [[45](#_ENREF_45)] |
|  | RORC | RAR-related orphan receptor C. | Breast Cancer | RORC expression is positively correlated with Foxp3 and IL-17A in synchronically increased Treg and Th17 cells in invasive ductal carcinoma of the breast which in turn is associated with tumor aggressiveness. [[46](#_ENREF_46)] |
|  | GYS2 | Glycogen Synthase 2 | Breast cancer | Involved in insulin signaling pathway and reported for breast cancer survival [[47](#_ENREF_47)] |
|  | RPL19P12 | Ribosomal protein L19 pseudogene 12 | Breast Cancer | Reported as additional reporter gene for HER2 molecular subgroup [[48](#_ENREF_48)] |
|  | AP1M1 | Adaptor Protein Complex AP-1 Mu-1 Subunit | Bladder Cancer | Reported as urine marker for detection of bladder cancer [[49](#_ENREF_49)] |
|  | GSTO2 | glutathione S-transferase omega 2 | [Barrett's adenocarcinoma](http://www.malacards.org/card/barretts_adenocarcinoma) | -omega class of Glutathione S-transferase  -reported that overexpression induces apoptosis and possible role in cell signalling [[50](#_ENREF_50), [51](#_ENREF_51)]  -disease association (Gene Cards) |
|  | MYOZ3 | Myozenin 3 | # | # |
|  | C13orf16 | chromosome 13 open reading frame 16 | # | # |
|  | EYA1 | Eyes absent homolog 1 | # | # |
|  | UFSP2 | UFM1-specific peptidase 2 | # | # |
|  | NOP2 | NOP2 nucleolar protein homolog | # | # |
|  | KIAA1737 | Uncharacterized protein KIAA1737 | # | # |
|  | HPS5 | Hermansky-Pudlak syndrome 5 | # | # |
|  | SCARNA20 | Small Cajal Body-Specific RNA 20 | # | # |
|  | KIAA0652 | KIAA0652 | # | # |
|  | C9orf71 | chromosome 9 open reading frame 71 | # | # |
|  | TTF2 | Transcription Termination Factor, RNA Polymerase II | # | # |
|  | C11orf73 | chromosome 11 open reading frame 73 | # | # |
|  | HBG1 | Hemoglobin, gamma 1 | # | # |
|  | TMC3 | transmembrane channel-like 3 | # | # |
|  | UGT2B10 | Uridine 5’-diphosphoglucuronosyltransferase | # | # |
|  | CNTD1 | Cyclin N-terminal domain-containing 1 | # | # |
|  | NTM | neurotrimin | # | # |
|  | KIAA1324L | KIAA1324-like | # | # |
|  | IGSF9 | Immunoglobulin Superfamily, Member 9 | # | # |
|  | C5orf62 | Chromosome 5 ORF 62; | # | # |

|  |
| --- |
|  |
|  |

1. Wang X, Tournier C: **Regulation of cellular functions by the ERK5 signalling pathway**. *Cellular signalling* 2006, **18**(6):753-760.

2. Rachagani S, Kumar S, Batra SK: **MicroRNA in pancreatic cancer: pathological, diagnostic and therapeutic implications**. *Cancer letters* 2010, **292**(1):8-16.

3. Lindgren D, Liedberg F, Andersson A, Chebil G, Gudjonsson S, Borg Å, Månsson W, Fioretos T, Höglund M: **Molecular characterization of early-stage bladder carcinomas by expression profiles, FGFR3 mutation status, and loss of 9q**. *Oncogene* 2006, **25**(18):2685-2696.

4. Tan X, Zhai Y, Chang W, Hou J, He S, Lin L, Yu Y, Xu D, Xiao J, Ma L: **Global analysis of metastasis‐associated gene expression in primary cultures from clinical specimens of clear‐cell renal‐cell carcinoma**. *International Journal of Cancer* 2008, **123**(5):1080-1088.

5. Cohen L, Cole SW, Sood AK, Prinsloo S, Kirschbaum C, Arevalo JM, Jennings NB, Scott S, Vence L, Wei Q: **Depressive symptoms and cortisol rhythmicity predict survival in patients with renal cell carcinoma: role of inflammatory signaling**. *PloS one* 2012, **7**(8):e42324.

6. Ricketts CJ, Morris MR, Gentle D, Brown M, Wake N, Woodward ER, Clarke N, Latif F, Maher ER: **Genome-wide CpG island methylation analysis implicates novel genes in the pathogenesis of renal cell carcinoma**. *Epigenetics* 2012, **7**(3):278-290.

7. Barabutis N, Schally Av: **Growth hormone-releasing hormone: extrapituitary effects in physiology and pathology**. *Cell Cycle* 2010, **9**(20):4110-4116.

8. Halmos G, Schally AV, Varga JL, Plonowski A, Rekasi Z, Czompoly T: **Human renal cell carcinoma expresses distinct binding sites for growth hormone-releasing hormone**. *Proceedings of the National Academy of Sciences* 2000, **97**(19):10555-10560.

9. Stephenson AJ, Smith A, Kattan MW, Satagopan J, Reuter VE, Scardino PT, Gerald WL: **Integration of gene expression profiling and clinical variables to predict prostate carcinoma recurrence after radical prostatectomy**. *Cancer* 2005, **104**(2):290-298.

10. Rae JM, Johnson MD, Cordero KE, Scheys JO, Larios JM, Gottardis MM, Pienta KJ, Lippman ME: **GREB1 is a novel androgen-regulated gene required for prostate cancer growth**. *The Prostate* 2006, **66**(8):886-894.

11. Ghosh MG, Thompson DA, Weigel RJ: **PDZK1 and GREB1 Are Estrogen-regulated Genes Expressed in Hormone-responsive Breast Cancer1, 2**. *Cancer research* 2000, **60**(22):6367-6375.

12. Ozen M, Creighton C, Ozdemir M, Ittmann M: **Widespread deregulation of microRNA expression in human prostate cancer**. *Oncogene* 2007, **27**(12):1788-1793.

13. Stolk JA, Jiang Y, Day CH, Klee JI, Zhang X, Dillon DC, Houghton RL, Harlan D, Reed SG, Xu J: **P704P, P712P, and P775P: A genomic cluster of prostate‐specific genes**. *The Prostate* 2004, **60**(3):214-226.

14. Black PC, Mize GJ, Karlin P, Greenberg DL, Hawley SJ, True LD, Vessella RL, Takayama TK: **Overexpression of protease-activated receptors-1,-2, and-4 (PAR-1, -2, and -4) in prostate cancer**. *The Prostate* 2007, **67**(7):743-756.

15. Kajihara H, Yamada Y, Kanayama S, Furukawa N, Noguchi T, Haruta S, Yoshida S, Sado T, Oi H, Kobayashi H: **Clear cell carcinoma of the ovary: Potential pathogenic mechanisms (Review)**. *Oncology reports* 2010, **23**(5):1193-1203.

16. Yan PY, Yu G, Tseng G, Cieply K, Nelson J, Defrances M, Zarnegar R, Michalopoulos G, Luo J-H: **Glutathione peroxidase 3, deleted or methylated in prostate cancer, suppresses prostate cancer growth and metastasis**. *Cancer research* 2007, **67**(17):8043-8050.

17. Bauerschlag DO, Ammerpohl O, Bräutigam K, Schem C, Lin Q, Weigel MT, Hilpert F, Arnold N, Maass N, Meinhold-Heerlein I: **Progression-free survival in ovarian cancer is reflected in epigenetic DNA methylation profiles**. *Oncology* 2011, **80**(1-2):12-20.

18. Helms MW, Kemming D, Contag CH, Pospisil H, Bartkowiak K, Wang A, Chang S-Y, Buerger H, Brandt BH: **TOB1 is regulated by EGF-dependent HER2 and EGFR signaling, is highly phosphorylated, and indicates poor prognosis in node-negative breast cancer**. *Cancer research* 2009, **69**(12):5049-5056.

19. Malecki M, Dahlke J, Haig M, Wohlwend L, Malecki R: **Eradication of Human Ovarian Cancer Cells by Transgenic Expression of Recombinant DNASE1, DNASE1L3, DNASE2, and DFFB Controlled by EGFR Promoter: Novel Strategy for Targeted Therapy of Cancer**. *J Genet Syndr Gene Ther* 2013, **4**(152):2.

20. Nikolova DN, Doganov N, Dimitrov R, Angelov K, Low S-K, Dimova I, Toncheva D, Nakamura Y, Zembutsu H: **Genome-wide gene expression profiles of ovarian carcinoma: Identification of molecular targets for the treatment of ovarian carcinoma**. *Molecular Medicine Reports* 2009, **2**(3):365-384.

21. Yoshihara K, Tajima A, Adachi S, Quan J, Sekine M, Kase H, Yahata T, Inoue I, Tanaka K: **Germline copy number variations in BRCA1‐associated ovarian cancer patients**. *Genes, Chromosomes and Cancer* 2011, **50**(3):167-177.

22. Sandoval J, Mendez-Gonzalez J, Nadal E, Chen G, Carmona FJ, Sayols S, Moran S, Heyn H, Vizoso M, Gomez A: **A Prognostic DNA Methylation Signature for Stage I Non–Small-Cell Lung Cancer**. *Journal of Clinical Oncology* 2013, **31**(32):4140-4147.

23. Fèvre-Montange M, Champier J, Durand A, Wierinckx A, Honnorat J, Guyotat J, Jouvet A: **Microarray gene expression profiling in meningiomas: differential expression according to grade or histopathological subtype**. *International journal of oncology* 2009, **35**(6):1395-1407.

24. Schmidt B, Liebenberg V, Dietrich D, Schlegel T, Kneip C, Seegebarth A, Flemming N, Seemann S, Distler J, Lewin J: **SHOX2 DNA methylation is a biomarker for the diagnosis of lung cancer based on bronchial aspirates**. *BMC cancer* 2010, **10**(1):600.

25. Naderi A, Teschendorff A, Barbosa-Morais N, Pinder S, Green A, Powe D, Robertson J, Aparicio S, Ellis I, Brenton J: **A gene-expression signature to predict survival in breast cancer across independent data sets**. *Oncogene* 2006, **26**(10):1507-1516.

26. Han H, Wang Y, Li X, Wang P-A, Wei X, Liang W, Ding G, Yu X, Bao C, Zhang Y: **Novel Role of NOD2 in Mediating Ca2+ Signaling Evidence From NOD2-Regulated Podocyte TRPC6 Channels in Hyperhomocysteinemia**. *Hypertension* 2013, **62**(3):506-511.

27. Lin Z-Y, Chuang W-L: **Genes responsible for the characteristics of primary cultured invasive phenotype hepatocellular carcinoma cells**. *Biomedicine & Pharmacotherapy* 2012, **66**(6):454-458.

28. Zhou X, Popescu NC, Klein G, Imreh S: **The interferon-α responsive gene< i> TMEM7</i> suppresses cell proliferation and is downregulated in human hepatocellular carcinoma**. *Cancer genetics and cytogenetics* 2007, **177**(1):6-15.

29. Hodgson JG, Yeh R-F, Ray A, Wang NJ, Smirnov I, Yu M, Hariono S, Silber J, Feiler HS, Gray JW: **Comparative analyses of gene copy number and mRNA expression in glioblastoma multiforme tumors and xenografts**. *Neuro-oncology* 2009, **11**(5):477-487.

30. Smith R, Owen LA, Trem DJ, Wong JS, Whangbo JS, Golub TR, Lessnick SL: **Expression profiling of EWS/FLI identifies< i> NKX2. 2</i> as a critical target gene in Ewing's sarcoma**. *Cancer cell* 2006, **9**(5):405-416.

31. Kaneda H, Arao T, Tanaka K, Tamura D, Aomatsu K, Kudo K, Sakai K, De Velasco MA, Matsumoto K, Fujita Y: **FOXQ1 is overexpressed in colorectal cancer and enhances tumorigenicity and tumor growth**. *Cancer research* 2010, **70**(5):2053-2063.

32. Neagu M, Constantin C, Tanase C, Boda D: **Patented biomarker panels in early detection of cancer**. *Recent patents on biomarkers* 2011, **1**:10-24.

33. Neeb A, Wallbaum S, Novac N, Dukovic-Schulze S, Scholl I, Schreiber C, Schlag P, Moll J, Stein U, Sleeman J: **The immediate early gene Ier2 promotes tumor cell motility and metastasis, and predicts poor survival of colorectal cancer patients**. *Oncogene* 2011, **31**(33):3796-3806.

34. Ghoshal K, Motiwala T, Claus R, Yan P, Kutay H, Datta J, Majumder S, Bai S, Majumder A, Huang T: **HOXB13, a target of DNMT3B, is methylated at an upstream CpG island, and functions as a tumor suppressor in primary colorectal tumors**. *PloS one* 2010, **5**(4):e10338.

35. BAYLIN S, CRIEKINGE W, SCHUEBEL K, COPE L, SUZUKI H, HERMAN J: **EARLY DETECTION AND PROGNOSIS OF COLON CANCERS**. In*.*: WO Patent 2,008,010,975; 2008.

36. Foster MC, Coresh J, Fornage M, Astor BC, Grams M, Franceschini N, Boerwinkle E, Parekh RS, Kao WL: **APOL1 Variants Associate with Increased Risk of CKD among African Americans**. *Journal of the American Society of Nephrology* 2013.

37. Bettermann K, Benesch M, Weis S, Haybaeck J: **SUMOylation in carcinogenesis**. *Cancer letters* 2012, **316**(2):113-125.

38. Spilka R, Ernst C, Mehta AK, Haybaeck J: **Eukaryotic translation initiation factors in cancer development and progression**. *Cancer letters* 2013, **340**(1):9-21.

39. Pardo LA, Stühmer W: **The roles of K+ channels in cancer**. *Nature Reviews Cancer* 2014, **14**(1):39-48.

40. Gerhardt J, Montani M, Wild P, Beer M, Huber F, Hermanns T, Müntener M, Kristiansen G: **FOXA1 promotes tumor progression in prostate cancer and represents a novel hallmark of castration-resistant prostate cancer**. *The American journal of pathology* 2012, **180**(2):848-861.

41. DeGraff DJ, Clark PE, Cates JM, Yamashita H, Robinson VL, Yu X, Smolkin ME, Chang SS, Cookson MS, Herrick MK: **Loss of the urothelial differentiation marker FOXA1 is associated with high grade, late stage bladder cancer and increased tumor proliferation**. *PloS one* 2012, **7**(5):e36669.

42. Habashy HO, Powe DG, Rakha EA, Ball G, Paish C, Gee J, Nicholson RI, Ellis IO: **Forkhead-box A1 (FOXA1) expression in breast cancer and its prognostic significance**. *European journal of cancer* 2008, **44**(11):1541-1551.

43. Bidwell BN, Slaney CY, Withana NP, Forster S, Cao Y, Loi S, Andrews D, Mikeska T, Mangan NE, Samarajiwa SA: **Silencing of Irf7 pathways in breast cancer cells promotes bone metastasis through immune escape**. *Nature medicine* 2012, **18**(8):1224-1231.

44. Shi Z, Derow CK, Zhang B: **Co-expression module analysis reveals biological processes, genomic gain, and regulatory mechanisms associated with breast cancer progression**. *BMC systems biology* 2010, **4**(1):74.

45. Dalgin G, Alexe G, Scanfeld D, Tamayo P, Mesirov J, Ganesan S, DeLisi C, Bhanot G: **Portraits of breast cancer progression**. *BMC bioinformatics* 2007, **8**(1):291.

46. Benevides L, Cardoso CR, Tiezzi DG, Marana HR, Andrade JM, Silva JS: **Enrichment of regulatory T cells in invasive breast tumor correlates with the upregulation of IL‐17A expression and invasiveness of the tumor**. *European journal of immunology* 2013.

47. Wei Z, Li H: **A Markov random field model for network-based analysis of genomic data**. *Bioinformatics* 2007, **23**(12):1537-1544.

48. Krijgsman O, Roepman P, Glas AM: **Means and methods for molecular classification of breast cancer**. In*.*: US Patent App. 13/546,755; 2012.

49. **Urine markers for detection of bladder cancer**. In*.*: EP Patent 2,436,779; 2012.

50. Wang L, Xu J, Ji C, Gu S, Lv Y, Li S, Xu Y, Xie Y, Mao Y: **Cloning, expression and characterization of human glutathione S-transferase Omega 2**. *International journal of molecular medicine* 2005, **16**(1):19.

51. McIlwain CC, Townsend DM, Tew KD: **Glutathione S-transferase polymorphisms: cancer incidence and therapy**. *Oncogene* 0000, **25**(11):1639-1648.
