# Supplementary figures and images for: Classification models for clear cell renal carcinoma stage progression, based on tumor RNAseq expression trained supervised machine learning algorithms
Source: BMC Proc. 2014 Oct 13;8(Suppl 6):S2. doi: 10.1186/1753-6561-8-S6-S2 (PMC4202178; doi:10.1186/1753-6561-8-S6-S2)

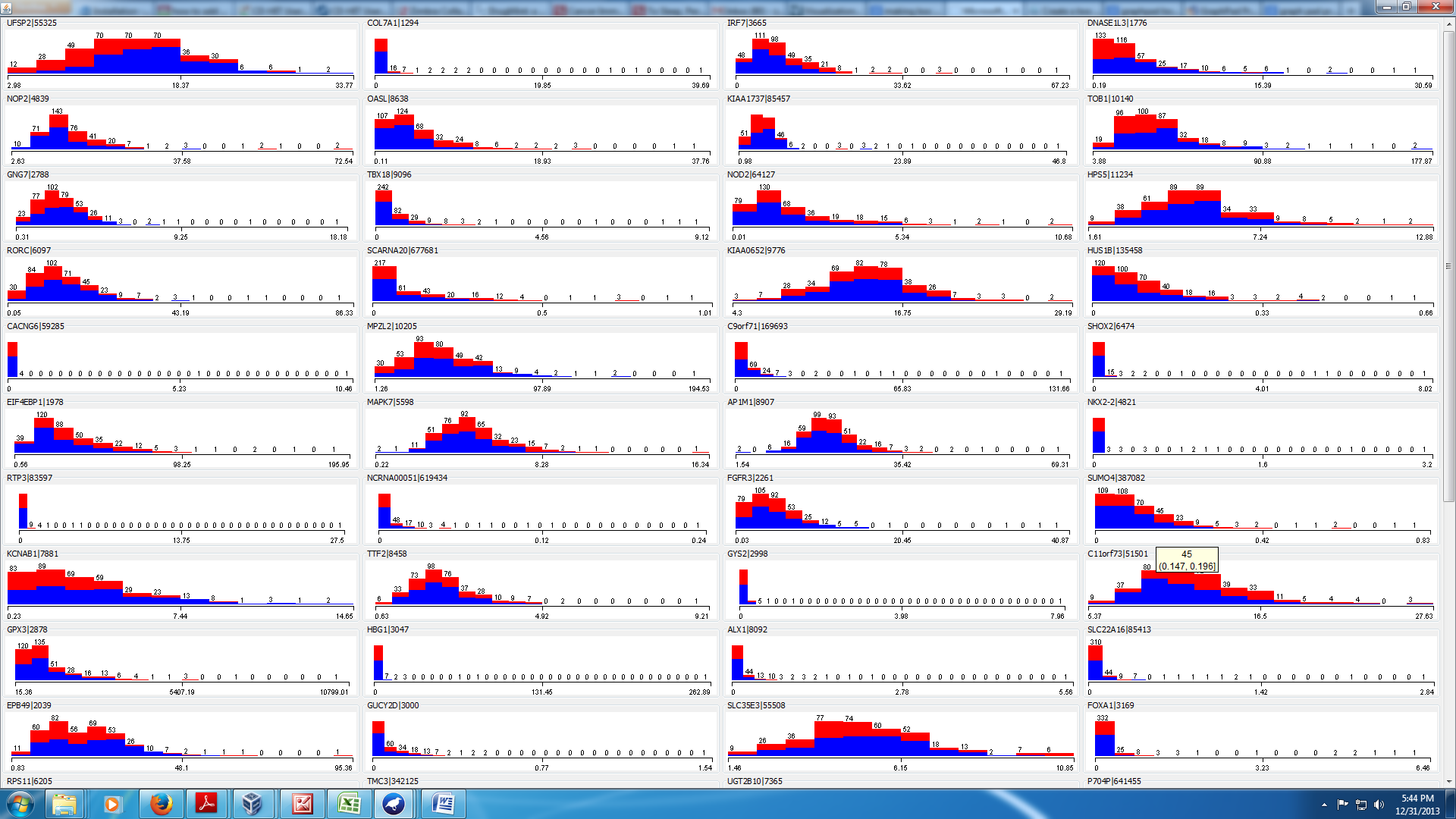

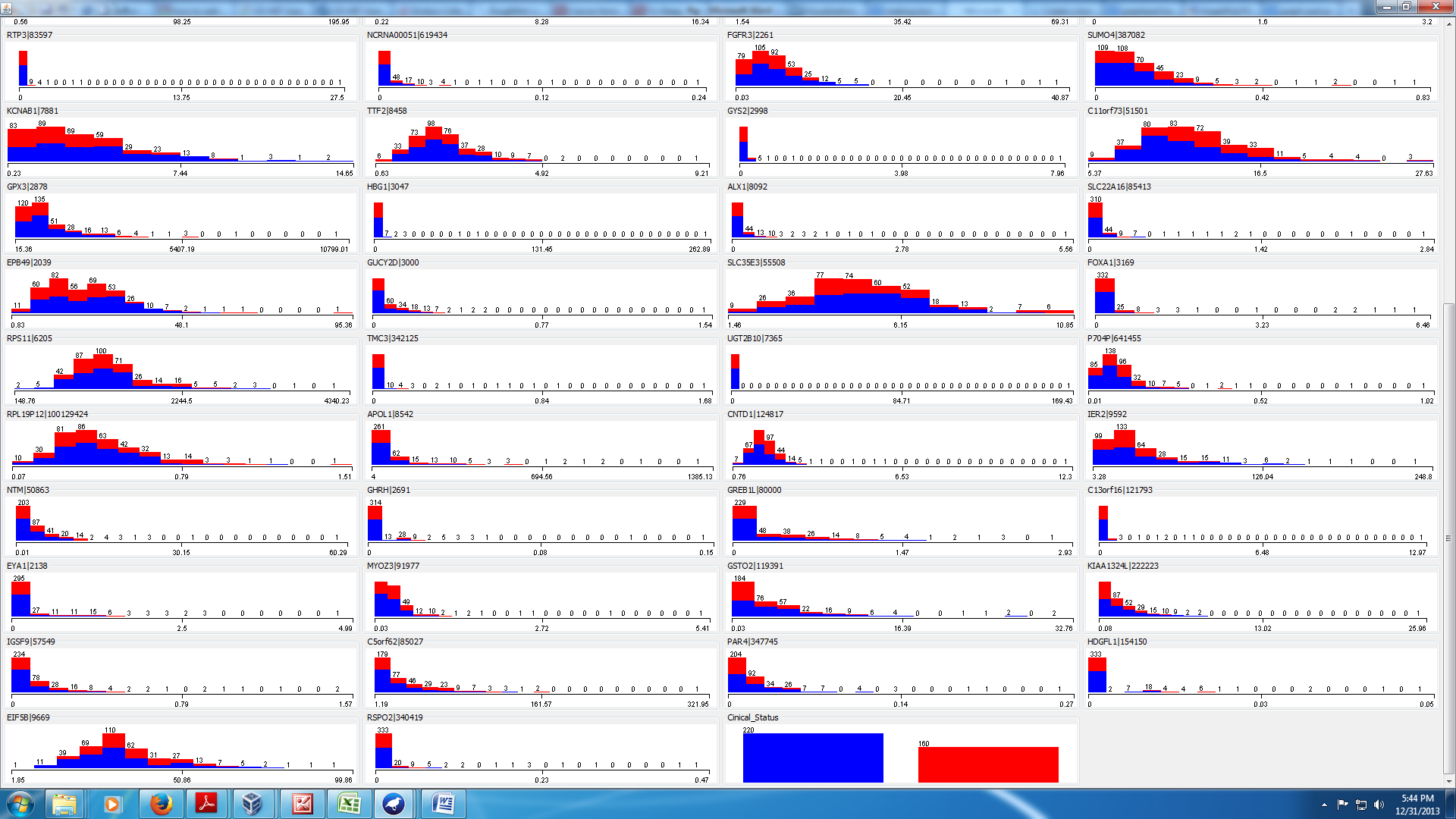

Supplement: Additional file 5 — Distribution plot of the expression values selected 62 genes. This file consists of expression value distribution plots generated by Weka Explorer of final 62 genes for the class label "Early Stage" and "Late Stage". The file is in .docx format and can be viewed using any document viewer like Microsoft Word. [file 1753-6561-8-S6-S2-S5.docx]
