# Supplementary material for: Classification models for clear cell renal carcinoma stage progression, based on tumor RNAseq expression trained supervised machine learning algorithms
Source: BMC Proc. 2014 Oct 13;8(Suppl 6):S2. doi: 10.1186/1753-6561-8-S6-S2 (PMC4202178; doi:10.1186/1753-6561-8-S6-S2)
Supplement: Additional file 6 — Analysis of the 62 selected genes for GO annotations - Biological Process; Oncoprint and Overall Survival Kaplan-Meier Estimate from cBioportal for Cancer Genomics. This file consists of 3 figures - Bar chart of GO biological process categories of the selected 62 genes; Screen Shot of Oncoprint for genomic alterations in selected 62 genes in ccRCC cases in cBioPortal for Cancer Genomics age distribution of the patients; and Survival Kaplan-Meier Estimate for selected 62 genes in ccRCC cases in cBioPortal for Cancer Genomics. The figures are in a Portable Document Format (PDF) and can be viewed with any standard PDF viewer. [file 1753-6561-8-S6-S2-S6.pdf]

Figure S5A: GO annotation for Biological Process by WebGestalt. (http://bioinfo.vanderbilt.edu/webgestalt/)

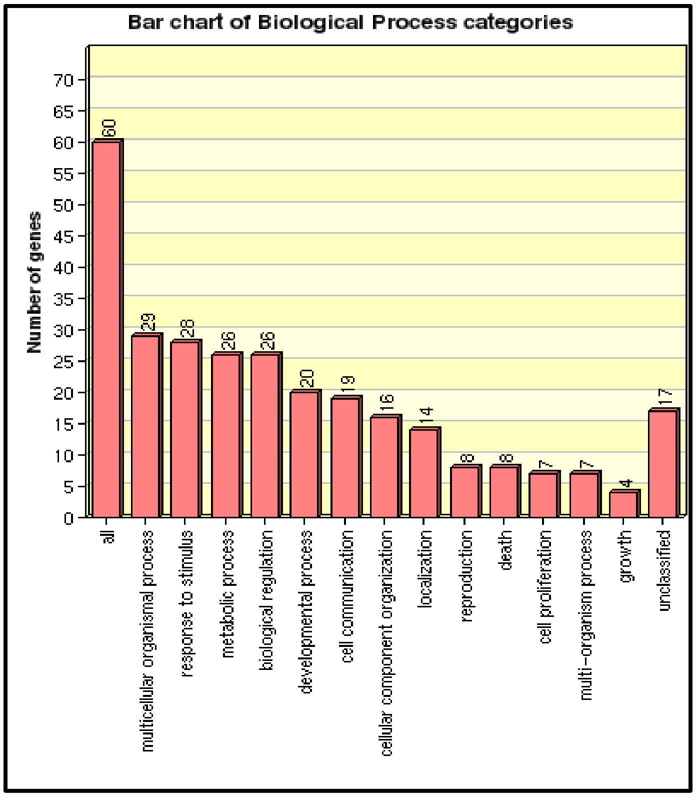

Figure S5B: Screen Shot of Oncoprint for genomic alterations in selected 62 genes in ccRCC cases in cBioPortal for Cancer Genomics.

(http://www.cbioportal.org/public-portal/)

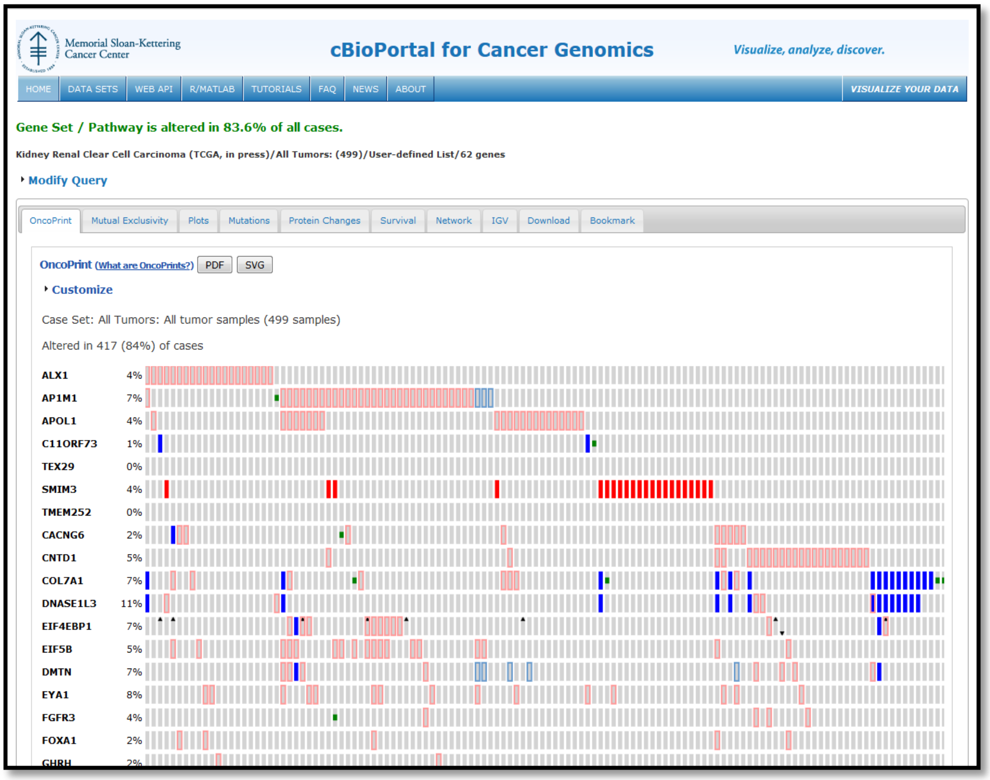

Figure S5C: Overall Survival Kaplan-Meier Estimate for selected 62 genes in ccRCC cases in cBioPortal for Cancer Genomics. (http://www.cbioportal.org/public-portal/)

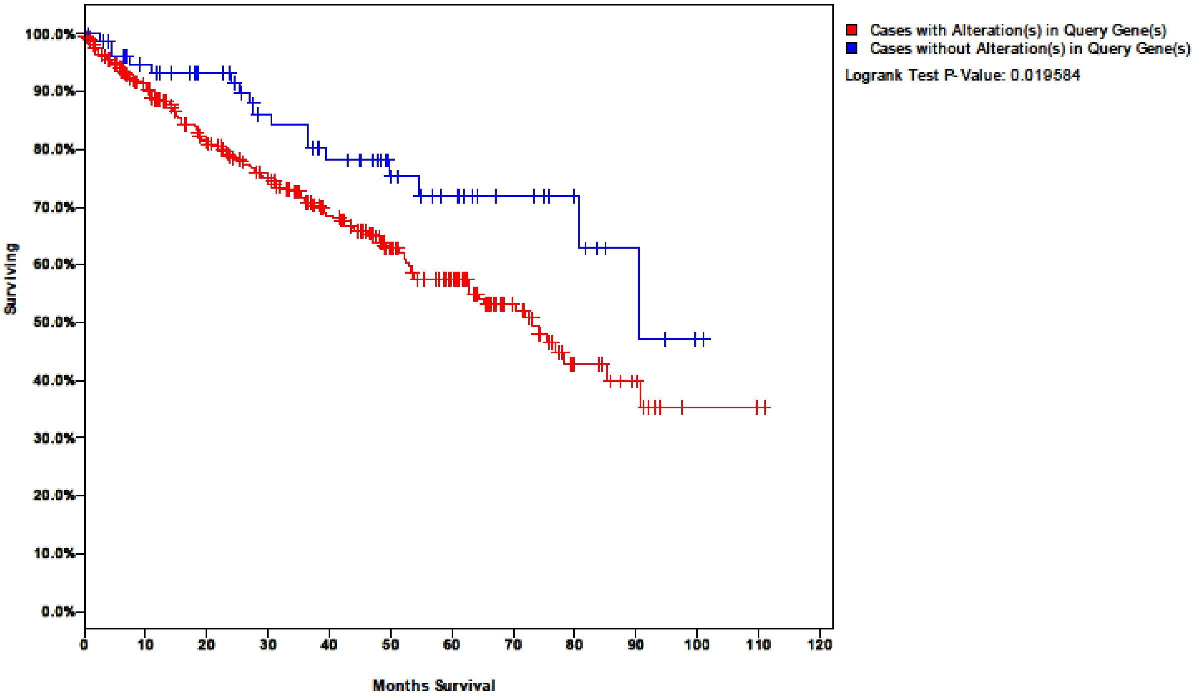

	#total cases	#cases deceased	median months survival
Cases with Alteration(s) in Query Gene(s)	364	130	73.17
Cases without Alteration(s) in Query Gene(s)	78	17	90.38
